# Sensitivity to Nonaccidental Configurations of Two-Line Stimuli

**DOI:** 10.1177/2041669517699628

**Published:** 2017-04-03

**Authors:** Jonas Kubilius, Charlotte Sleurs, Johan Wagemans

**Affiliations:** Brain & Cognition, KU Leuven, Belgium

**Keywords:** Perceptual organization, nonaccidental properties, configural processing, geons

## Abstract

According to Recognition-By-Components theory, object recognition relies on a specific subset of three-dimensional shapes called *geons*. In particular, these configurations constitute a powerful cue to three-dimensional object reconstruction because their two-dimensional projection remains viewpoint-invariant. While a large body of literature has demonstrated sensitivity to changes in these so-called nonaccidental configurations, it remains unclear what information is used in establishing such sensitivity. In this study, we explored the possibility that nonaccidental configurations can already be inferred from the basic constituents of objects, namely, their edges. We constructed a set of stimuli composed of two lines corresponding to various nonaccidental properties and configurations underlying the distinction between geons, including collinearity, alignment, curvature of contours, curvature of configuration axis, expansion, cotermination, and junction type. Using a simple visual search paradigm, we demonstrated that participants were faster at detecting targets that differed from distractors in a nonaccidental property than in a metric property. We also found that only some but not all of the observed sensitivity could have resulted from simple low-level properties of our stimuli. Given that such sensitivity emerged from a configuration of only two lines, our results support the view that nonaccidental configurations could be encoded throughout the visual processing hierarchy even in the absence of object context.

## Introduction

Which principal factors lead to an efficient organization of a visual scene into objects and backgrounds? Since the early days of experimental psychology, Gestalt grouping laws, such as proximity, similarity, and good continuation, have offered a powerful means to understand and predict the structure of our percepts (Wagemans, Elder, et al., 2012; Wagemans, Feldman, et al., 2012; Wertheimer, 1923). Based on these grouping principles, separate elements and parts in an image can be grouped together into larger clusters or coherent wholes in the presence of clutter or noise.

Gestalt grouping principles are not the only basis to perceive structure in a scene though. For example, observing that two elements are parallel is important because this relationship remains constant from nearly any viewpoint. If the goal is to perceive the three-dimensional (3D) structure of an object or to recognize its identity, such viewpoint-independent relations can be very informative. Although it remains true that an image can result from infinitely many different 3D scenes, to find a particular type of regularity in the image for non-corresponding regularities in the world would be quite accidental. Indeed, it usually only happens with one specific viewpoint. Under the assumption of a generic viewpoint, therefore, these image regularities usually signal corresponding scene regularities. For this reason, these image regularities are called *nonaccidental properties* (Lowe, 1985). Examples of nonaccidental properties (NAPs) include curvilinearity, collinearity, cotermination, parallelism, and skew-symmetry. In contrast, observing that the two parts intersect at a particular angle is much less informative since the projected angle on the retina is viewpoint-dependent (e.g., Willems & Wagemans, 2000).

According to the Recognition-By-Components (RBC) theory (Biederman, 1987), these NAPs play an essential role in quickly deriving the essential building blocks of objects and interpreting our surroundings in terms of objects. In particular, Biederman proposed that object recognition relies on a small set of 3D geometric primitives called *geons* that are derived from nonaccidental edge configurations. For example, a brick and a pyramid differ in the parallelism of the edges and are thus rarely confused in their 2D projection to the eye, despite changes in viewpoint. Conversely, a brick and a cube do not differ in terms of nonaccidental features and thus cannot always be distinguished solely based on their 2D projections.

Biederman and colleagues have accumulated an impressive body of evidence that the primate visual system indeed is sensitive to NAPs. For example, Kayaert, Biederman, and Vogels (2003) compared neural responses in the monkey inferotemporal cortex by presenting stimuli differing from a base stimulus (e.g., a pyramid) either in a NAP (resulting in a brick) or a metric property (MP) equally distant from the base stimulus (resulting in a shallower pyramid). They found that neurons responded more vigorously to objects that differed in NAPs than when they differed in MPs. Similarly, by measuring accuracy in a match-to-sample task, Amir, Biederman, and Hayworth (2012) found that participants were more sensitive behaviorally to both 2D and 3D geons differing in a wide range of NAPs (see also Todd et al., 2014). This sensitivity to NAPs appears to be a very general property of the visual system, observed in infants (Kayaert & Wagemans, 2010), children (Amir, Biederman, Herald, Shah, & Mintz, 2014; Ons & Wagemans, 2011), non-urban cultures (Biederman, Yue, & Davidoff, 2009), and non-mammalian species (Gibson, Lazareva, Gosselin, Schyns, & Wasserman, 2007; Lazareva, Wasserman, & Biederman, 2008; Peissig, Young, Wasserman, & Biederman, 2000). Neural measurements in monkeys pointed to the inferotemporal cortex as a possible locus of such sensitivity (Kayaert et al., 2003; Kayaert, Biederman, Op de Beeck, & Vogels, 2005; Vogels, Biederman, Bar, & Lorincz, 2001) and more recently the shape-selective lateral occipital cortex (LOC) in humans has also been shown to respond to changes in NAPs (Amir, Biederman, & Hayworth, 2011; Kim & Biederman, 2012). Finally, NAPs have also been claimed to play an important role in scene recognition. Walther and Shen (2014) and Choo and Walther (2016) showed that at least some NAPs, namely, junctions and junction angles, might also underlie scene categorization by humans.

Here we demonstrate that sensitivity to NAPs holds even in the absence of object or shape context. We constructed a set of stimuli composed of two line segments only, corresponding to the nonaccidental configurations in the original geons. Even in these simple displays we found a pronounced sensitivity to NAPs, indicating that the computation of nonaccidental properties is not exclusive to object processing and instead reflects generic image processing mechanisms in the visual system.

## Methods

### Participants

Ten students from KU Leuven participated in the experiment (age: 21–23; males: 3, females: 7) and were paid €8 for their participation. Ten additional students from KU Leuven (9 participants of age less 20, one between 20 and 29; males: 1, females: 9) participated in a replication of this experiment and received course credit for their participation. All participants had normal or corrected-to-normal vision and provided a written informed consent. The experiments were approved by the ethical committee of the Faculty of Psychology and Educational Sciences.

### Stimuli

**Figure 1.**
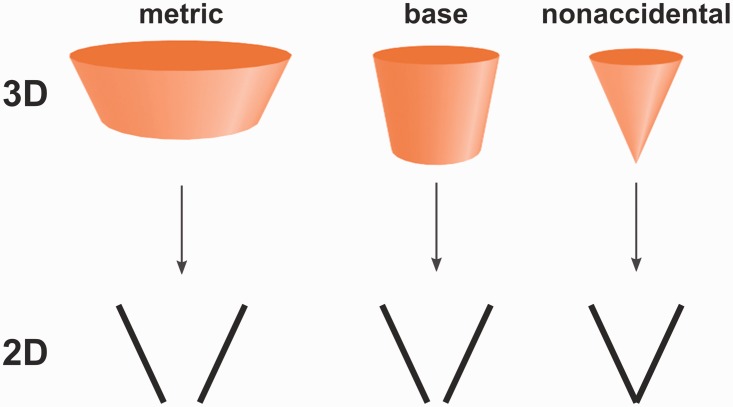
An example how geons were translated into two-line stimuli.

**Figure 2.**
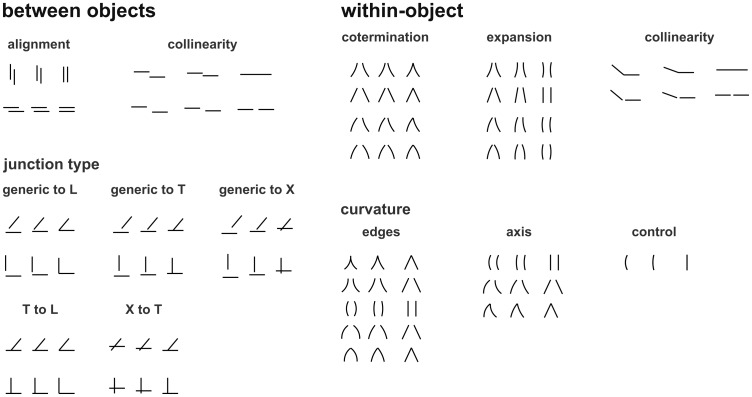
Examples of stimuli for each of 13 conditions in the experiment. In each triplet, the middle stimulus is the base stimulus, the one on the left is its metric variant (MP), and the one on the right is the nonaccidental variant (NAP). Note that in the actual experiment we had many more exemplars for each condition (78 triplets in total), constructed by mirroring the shown stimuli upside-down or left-right.

Our aim was to investigate whether the visual system was sensitive to nonaccidental configurations even when no object context was provided. We therefore translated geons and configurations of geons used in various experiments by Biederman and his colleagues into stimuli composed of two line segments only (Figure 1; Amir et al., 2012; Kim & Biederman, 2012), resulting in 12 experimental conditions (Figure 2):

1. NAPs between objects:

- Alignment: whether objects are aligned or not.
- Collinearity: whether objects are on the same line or not.
- Junction type: the kind of junction that two objects are forming:

- Generic to L
- Generic to T
- Generic to X
- T to L
- X to T

1. NAPs within objects:

- Cotermination: whether edges of an object are coterminating or not.
- Expansion vs. constant: whether edges of an object are at a constant distance or expanding
- Collinearity: whether edges of an object are collinear or not
- Curvature:

- Edges: whether edges of an object are straight or curved
- Axes: whether object’s axis is straight or curved

We also had an additional condition where the stimulus consisted of a single line segment and its curvature was manipulated. This condition served as a control for the two curvature conditions where participants could discriminate between the variants not based on the nonaccidental configuration but the curvature alone.

Note that not all NAPs defining geons could be translated to two-line configurations, such as a straight versus a curved cross section (Dickinson & Biederman, 2014). Moreover, it is not exactly clear whether the junction configurations truly correspond to nonaccidental configurations. However, we included them for completeness, since *occlusion* was considered a nonaccidental property in Kim and Biederman (2012). Furthermore, it is possible that observers treat these junction stimuli not as two separate objects but rather as one, and thus, the nonaccidental relation holds in this two-dimensional context.

For each stimulus, which we refer to as the base stimulus, two variants were created. The nonaccidental variant featured a very similar configuration that differed from the base in terms of a single nonaccidental property. In contrast, the metric variant had the same configuration as the base but differed to the same extent as the nonaccidental variant but in the opposite direction such that there was no change in nonaccidental properties.

### Setup

Experiments and analyses were coded in Python 2.7 using PsychoPy (Peirce, 2007, 2009), psychopy_ext (Kubilius, 2014), pandas, and statsmodels packages (source code available at https://bitbucket.org/qbilius/twolines).

**Figure 3.**
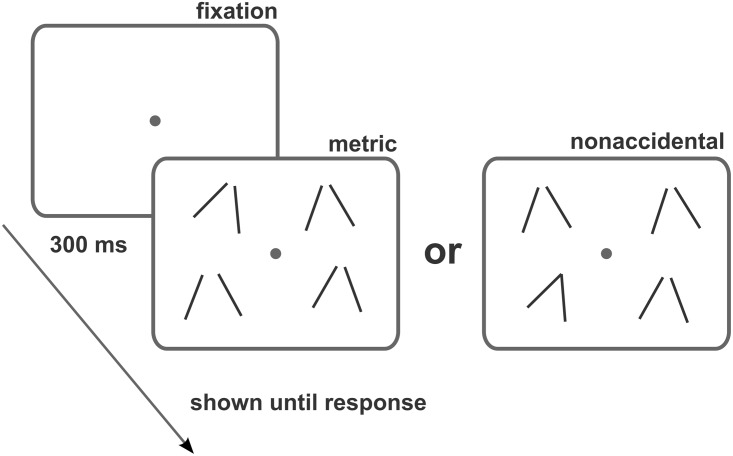
Experimental design. At each trial, participants were presented with four stimuli and had to indicate which one was different. In half of the trials, the odd stimulus differed from the rest in a nonaccidental change of configuration. In the other half, the odd stimulus was identical to the other stimuli in terms of its nonaccidental properties but differed in some metric property (e.g., angle) to the same amount as its nonaccidental counterpart. Note that in the actual experiment the stimuli were white and were presented on a gray background.

A trial was initiated by a key press. The participants saw a central fixation spot for 300 ms, followed by the onset of four stimuli, presented in the four quadrants of the display (Figure 3), modeled after Pomerantz, Sager, and Stoever (1977). Three of these stimuli were identical, while the remaining one (the target) was different, and participants were instructed to indicate via a key press as quickly and as accurately as possible which one of the four quadrants contained the target stimulus. The target was either the metric or the nonaccidental variant, and the three distractors were then the base stimuli, or the target was the base stimulus and the distractors were either three identical metric or nonaccidental variants. All possible combinations were tested only once, resulting in 1,248 trials in total, 78 (stimuli types) × 2 (metric vs. nonaccidental variant) × 2 (target vs. distractor) × 4 (target positions).

The stimuli subtended 3° in visual angle and were presented 5° away from the central fixation spot. The gap between the centers of the two line segments was approximately 1.5°. To make the task more challenging and avoid symmetry effects common in Pomerantz et al. (1977) displays, in each trial random jitter was added to the position (within ± .25°) and orientation (within ± 5°) of each stimulus independently. Trials were presented in a random order (conditions were interleaved). The experiment lasted approximately an hour.

## Results

**Figure 4.**
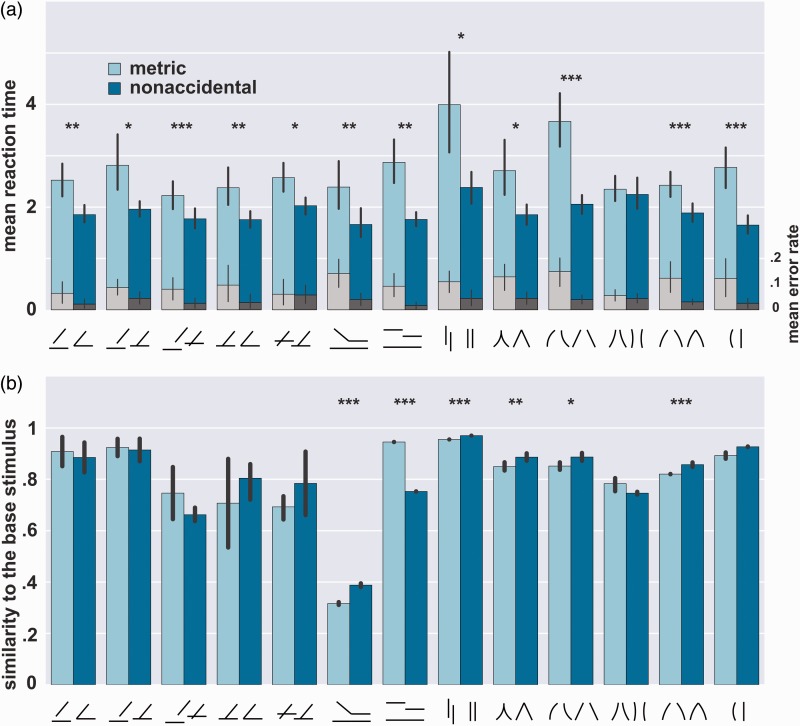
(a) Average response times per condition (blue) and average error rate (gray). Error bars denote the standard errors of the mean across participants (*n* = 10). *denotes *p*-value significant at α-level .05 for reaction times, **denotes *p*-value significant at α-level .01, ***denotes *p*-value significant at α-level .001 (after the Bonferroni correction). (b) Cosine similarity of metric and nonaccidental stimuli to the base stimulus as measured by GaborJet model outputs. Error bars denote the standard errors for the mean across stimuli of the same kind. Significance levels are indicated as in panel (a).

To investigate the effects of NAPs, we computed mean reaction time per stimulus condition (Figure 4). Note that typically reaction time measures are not distributed normally and thus computing mean reaction times per participant might lead to a poor estimate of the true reaction time. After a graphical inspection that normality was indeed violated, we computed the median reaction time per participant, which was used to compare reaction times to the nonaccidental and metric variants across participants. Bonferroni correction was applied to account for multiple testing.

We found that in almost all conditions, participants detected nonaccidental variants faster than their metric counterparts (Figure 4(a) and Table 1). The only condition that did not exhibit a statistically reliable effect was the expansion versus constant condition (*t*(9) = 1.82, *p* = .051). We reasoned that the two line segments might have appeared so close together in the metric variant that participants perceived them as coterminating, which is an undesirable nonaccidental change. To test if this was the case, we tested 10 additional participants to perform the task again but this time with a slightly larger gap between the two lines (2.25°). Moreover, to maximize the chances of finding any difference, we presented each condition in a separate block, so that participants would try just as hard for easy as for hard conditions. In this experiment, we found that all conditions nonaccidental changes were detected reliably faster that metric. (It should be noted however that the generic to T condition resulted in *p* = .005, which does not survive our strict Bonferroni correction criterion.)

**Table 1.** A Related-Samples One-Tailed *t* Test Results for Each Condition.

Condition	*t*(9)	*p*
Generic to L	5.83	<.001
Generic to T	3.79	.002
Generic to X	7.87	<.001
T to L	4.62	.001
X to T	4.06	.001
Collinearity by angle	4.73	.001
Collinearity by position	5.79	<.001
Alignment	3.67	.003
Curvature edges	4.33	.001
Curvature axis	8.17	<.001
Expansion vs constant	1.82	.051
Cotermination	7.56	<.001
Curvature control	7.52	<.001

Similar, albeit weaker, trends were found when accuracy was analyzed (Figure 4(a), gray bars). Since accuracy differences were likely influenced by ceiling effects (on average, participants reached 90% on metric changes and 97% on the nonaccidental ones), we did not analyze these effects any further.

We further asked if the observed effects for curvature in conditions curvature edge and curvature axis conditions were due to configural sensitivity per se or resulted solely from participants’ sensitivity to curvature in a single line (curvature control condition). To address this question, we performed a repeated-measures analysis of variance. We found no significant difference in the effect of distance (NAP vs. MP) between curvature edge and curvature control conditions (*F*(1,9) = .124, *p* = .727). In contrast, the effect of distance (NAP vs. MP) was significantly stronger between curvature axis and curvature control conditions (*F*(1,9) = 11.66, *p* = .002). These observations held in the replicated data as well, albeit less robustly (*F*(1,9) = 1.54, *p* = .223 and *F*(1,9) = 5.50, *p* = .025, respectively). Therefore, participants could have relied on judging the curvature of single line in the curvature edge condition, but not in the curvature axis condition where the configural information between the two lines disproportionately influenced participants’ decisions.

Finally, we asked if this pattern of results could be due to some low-level differences between stimuli that are not related to nonaccidentalness per se? Although we parametrically matched the distances of metric and nonaccidental variants from the base, it is still possible that a difference exists when the actual images of stimuli are processed by simple Gabor filters found in the visual area V1. Thus, if nonaccidental variants are found to be less similar to the base stimulus than the metric variant is to the base stimulus, any difference observed behaviorally could potentially result from the confounding low-level differences in stimuli. In contrast, if no difference is found, any behavior difference is more likely to stem from features computed later on in the visual system.

We therefore quantified the difference between the nonaccidental and metric variants using the GaborJet model (Lades et al., 1993), a common approach used by Biederman and colleagues to equate metric and nonaccidental variants. In a nutshell, this model computes V1-like features of each stimulus and a similarity is estimated using the one minus the cosine difference between these feature vectors, as described by Lades et al. (1993).

For our stimulus set, we found that all but one stimulus were properly matched or the similarity between the nonaccidental and the base stimulus was even larger than between the metric and the base one (Figure 4(b)). We also found that the Pearson correlation between this model and human reaction times (using nonaccidental minus metric) across all 78 stimulus triplets was only about −.14 (two-tailed *p* = .23). Overall, it is unlikely that the behaviorally observed differences resulted from simple low-level differences in stimuli.

## Discussion

Taken together, we demonstrated that the participants were sensitive to various nonaccidental configurations, even in the absence of object information. Unlike previous studies, here we showed that the visual system is sensitive to even the most basic form of nonaccidental configurations, composed of merely two lines. While some of these configurations might result from confounding changes in nonaccidental configurations (curvature edges, between-object collinearity), overall we found that the visual system is sensitive to even the most basic form of nonaccidental configurations, composed of merely two lines. These results are consistent with earlier theoretical, behavioral, and neural studies that reported sensitivity to the regularity in configurations of two-line stimuli (Feldman, 1997, 2007; Kubilius, Wagemans, & Op de Beeck, 2014a).

Based on these findings, it is possible that the encoding of configural information occurs as a default computation during the visual information processing. More specifically, nonaccidental relations between primitive shape features, such as edges, angles, and curves, might already be detected early on and communicated to the next processing stages even prior to object-centered visual processing and even in the absence of object recognition tasks. Notice that this suggestion reveals a broader range of configural information encoding than proposed by earlier studies where only angles and curved segments have been shown to be encoded (Ito & Komatsu, 2004; Pasupathy & Connor, 1999). It is worth mentioning however that to some extent our results could also be interpreted as reflecting not just any nonaccidental changes but rather changes in symmetry. Consistent with this view, higher visual areas have shown sensitivity to symmetry (Bertamini & Makin, 2014).

How early could these configurations be computed? Our GaborJet simulations that try to capture the basic processing in the visual area V1 imply that it is not likely to be the source of this computation. Instead, we suggest that truly configural processing might be required where the outputs of different kinds of simple cells (e.g., selective for different orientations or spatial frequencies) are combined. This idea is consistent recent demonstrations that primate visual area V2 computes summary statistics of edge-based responses (Freeman & Simoncelli, 2011; Freeman, Ziemba, Heeger, Simoncelli, & Movshon, 2013). Such summary statistics might be sufficient to reflect differences between metric and nonaccidental property (see also Kubilius, Wagemans, & Op de Beeck, 2014b, for a broader discussion of summary statistics computations in the visual cortex). Future studies could explore this possibility in depth.

On the other hand, in a similar two-line stimuli setup, Kubilius et al. (2014a) only observed sensitivity to these configurations in human lateral occipical cortex (LOC) but not earlier. Given that previous studies using three-dimensional geons consistently reported LOC or monkey IT (Kayaert et al., 2003) being sensitive to geon properties, our results indicate a possibility that LOC computes configural information between edges in addition to comparing full surface-based representations or matching to geon templates.

Finally, our findings are consistent with recent computer vision studies that demonstrated that a robust sensitivity to NAPs can emerge even without training explicitly for nonaccidental feature processing. Parker, Reichert, and Serre (2015) showed that a hierarchical model HMAX enhanced with a temporal continuity rule also develops a sensitivity to NAPs by merely observing videos of slowly rotating objects. A similar sensitivity is also present in deep convolutional neural networks that are optimized for object recognition and that are currently our best models of visual processing in the primate visual system (Kubilius, Bracci, & Op de Beeck, 2016; Rajalingham et al., 2015; Yamins et al., 2014). These computational studies indicate that the sensitivity to NAPs might not even rely on any explicit coding of nonaccidental properties but instead emerge as a result of the system absorbing statistical regularities from its visual inputs.

## Statement of Open Science

For maximal transparency, this research was carried out using as many free and open source software tools as possible, including GNU/Linux, Python (PsychoPy, psychopy_ext, pandas, statsmodels, and their dependencies), Mercurial (hg), Inkscape, and Scribus. Full source code and data of the study are available at https://bitbucket.org/qbilius/twolines.
